# Complications and survival after Ivor Lewis esophagectomy

**DOI:** 10.1186/s12885-026-16447-8

**Published:** 2026-07-01

**Authors:** A. Abu Hejleh, J. Lemties, I. Kreutzer, N. M. Wirsik, D. T. Krauss, S. Torabi, J-O Jung, W. Schröder, H. A. Schlößer, T. Schmidt, H. F. Fuchs, C. J. Bruns, L. M. Schiffmann

**Affiliations:** 1 Department of General, Visceral, Thoracic and Transplantation Surgery, Faculty of Medicine and University Hospital Cologne, University of Cologne, Cologne, Germany; 2 Department of Anesthesiology and Intensive Care Medicine, Medical Faculty of Cologne University, University Hospital of Cologne, Cologne, Germany; 3 Department of General, Visceral and Oncological Surgery, University Hospital Wuppertal, University of Witten-Herdecke, Wuppertal, Germany

**Keywords:** Survival, Complications, Esophagectomy, Ivor Lewis, Anastomotic leak

## Abstract

**Background:**

Esophagectomy (OE) remains a complex procedure with a considerable risk of severe postoperative complications. There is conflicting evidence if major postoperative complications impair long-term oncological survival. This retrospective study was designed to retest the hypothesis that complications after OE impact on long-term survival in a large and highly standardized cohort of esophageal cancer patients.

**Materials and methods:**

733 patients who underwent Ivor Lewis esophagectomy (IL-OE) for cancer from 2016 to 2021 at our tertiary center were analysed from a prospectively maintained database. Postoperative complications were correlated to overall survival.

**Results:**

Neither occurrence of major complications ≥ Clavien-Dindo (CD) IIIB (Median OS not reached in both groups, *p* = 0.45, HR 1.12) nor AL (Median OS not reached for no AL vs. 50 months for AL, *p* = 0.49, HR 0.96) nor pulmonary complications (Median OS not reached in both groups, *p* = 0.61, HR 0.76) had an impact on overall survival. However, the necessity for postoperative readmission to ICU (reaICU) had a significant impact on overall survival (Median OS not reached for no reaICU vs. 40 months for reaICU, *p* = 0.037, HR 1.54). Mean follow up was 27 months.

**Conclusion:**

Among all variables, only ICU readmission significantly affected overall survival. Postoperative complications and in particular anastomotic leakage after IL-OE, per se, may have a less significant impact on overall survival than previously anticipated. This might be due to a highly sufficient management of complications that progressively avoids requirement of severe septic complications and intensive care treatment.

**Supplementary Information:**

The online version contains supplementary material available at 10.1186/s12885-026-16447-8.

## Introduction

Esophagectomy (OE) for esophageal cancer or cancer of the esophagogastric junction remains a highly complex and demanding surgical procedure with an overall complication rate of 59% [1]. Despite advances in multimodal treatment protocols, surgical techniques and perioperative care, the procedure still comes along with life threatening complications resulting in considerable mortality rates [2]. To date, data on the impact of major complications on long-term survival and quality of life remains inconsistent and contradicting. Several studies linked postoperative complications, particularly anastomotic leakage (AL) and cardiopulmonary complications with a reduced long-term survival, regardless of patient and tumor characteristics or surgical technique [3–5]. This conclusion contrasts with large study cohorts from single high-volume centers demonstrating that long-term survival following OE is unaffected by complications, regardless of their severity [6, 7].

Among major complications AL remains one of the most challenging and severe complications after OE. It contributes to a prolonged hospital stay due to required interventional procedures or reoperation and is associated with increased cancer recurrence rates leading to poorer quality of life [8]. The incidence ranges from 11.4 to 21.2% wth a leak-associated mortality between 7.2 and 35% [9, 10]. Tis wide range is affected by factors such as surgical technique, anastomosis location, and patient-specific characteristics. Until now, the existing evidence regarding the effect of AL on long-term survival following OE remains likewise ambiguous [11, 12]. A significant distinction highlighted in the current literature is the volume effect in esophageal surgery. The impact of anastomotic leakage on the long-term survival depends on whether the patient received surgery in a high or low volume center. Recent studies indicate that AL following esophagectomy is significantly associated with reduced long-term survival. Notably, this adverse effect was significant in low-volume centers but did not apply for high-volume centers [3, 13].

Pulmonary complications (PC), including pneumonia, pleural effusion, or respiratory insufficiency, have been identified as independent predictors of poor outcomes with their adverse impact on short-term outcomes already documented by several study groups [14, 15]. However, the precise impact of PC on long-term survival remains a topic of debate. While numerous studies concluded that pulmonary infections are linked to unfavorable long-term outcomes in patients undergoing OE, others showed no association between PC and reduced long-term survival [16–18].

This study aims to investigate the impact of the most common complications after Ivor Lewis esophagectomy on overall survival in a highly standardized patient cohort from a high-volume center.

## Methods

Seven hundred thirty-three patients who underwent IL-OE for squamous cell carcinoma or adenocarcinoma of the esophagus or esophagogastric junction between January 2016 and December 2021 at the Department of General, Visceral, Thoracic and Transplantation Surgery, University Hospital of Cologne were included in this study. Data were retrospectively analysed from a prospectively maintained institutional database. Survival data were gathered during postoperative cancer surveillance through various methods, such as direct patient contact, correspondence by mail, telephone, communication with general practitioners, and review of central civil registers. The analysis was conducted in compliance with the guidelines defined by the Institutional Ethics Committee of the University Hospital of Cologne.

Diagnosis and primary staging of disease were performed using a combination of esophagogastroduodenoscopy with biopsy, endoscopic ultrasound, and spiral contrast-enhanced computer tomography of the thorax and abdomen. The cases were then reviewed in a multidisciplinary tumor conference to determine the most appropriate therapeutic options.

The standard surgical procedure was an IL-OE consisting of an abdominal phase and a right transthoracic en bloc esophagectomy with two-field lymphadenectomy of mediastinal and abdominal lymph nodes. Reconstruction was performed using an intrathoracic esophagogastrostomy. Surgical techniques included open approaches, hybrid resections (laparoscopic abdominal phase followed by open right thoracotomy), or total minimally invasive procedures. Alternative approaches, such as the three-stage McKeown esophagectomy and transhiatal gastrectomy, were excluded from this study.

The primary outcome in this study was overall survival in relation to the occurrence of any postoperative complication. Postoperative complications were monitored through routine daily clinical examinations and regular laboratory assessments. In the event of clinical or laboratory deterioration, esophagogastroduodenoscopy was performed to evaluate for anastomotic leakage. This was followed by computed tomography of the thorax and abdomen to assess for mediastinitis, pneumonia, seropneumothorax, or other surgical or pulmonary complications. Overall survival was defined as the period starting at 90 days from the date of surgery to either death or last follow-up. Complications were recorded and categorized according to the Clavien-Dindo (CD) classification for all in-hospital complications [19]. Minor complications were defined as CD I-IIIA, major complications were specified as CD ≥ IIIB. In addition to the overall complication rate, the study separately analysed the most significant complications following OE reported in literature, anastomotic leakage, and pulmonary complication, in relation to the primary endpoint of overall survival.

Pulmonary complications included pneumonia, respiratory insufficiency with ventilation > 72 h, pleural effusion drainage, tracheostomy and atelectasis with bronchoscopy.

Anastomotic leakage was defined in accordance with the Esophagectomy Complications Consensus Group (ECCG) as a ‘full-thickness gastrointestinal defect involving esophagus, anastomosis, staple line, or conduit irrespective of presentation or method of identification’ [20]. Pathological tumor and lymph node stage was defined using the 7th edition of the Union for International Cancer Control (UICC).

Initial admission to the intensive care unit (ICU) is the standard postoperative care, with a typical ICU stay of two days following hybrid procedures and one day after robot-assisted minimally invasive esophagectomy (RAMIE) or minimally invasive esophagectomy (MIE). ICU readmission criteria are assessed individually, based on the severity of identified complications and was strictly defined as a secondary transfer back to the ICU after initial discharge to the normal surgical ward. The surgical team primarily decides on ICU readmission in response to single- or multi-organ failure. Readmission to ICU may also be required in specific cases due to factors like significant comorbidities or non-surgical adverse events. To evaluate the impact of ICU readmission on postoperative outcomes, the overall cohort was divided into two groups (no readmission to ICU (no reaICU) vs. readmission to ICU (reaICU)). These groups were then compared in terms of postoperative complications.

Statistical analysis was performed using the open-source library Python in version 3.12.4. Postoperative complications were correlated to overall survival. Overall survival was estimated using the Kaplan-Meier analysis. Gehan-Breslow-Wilcoxon test, log rank testing and Chi-square test were used to compare categorical data starting at 90 days postoperative. Covariates for the multivariable Cox proportional hazards model were selected a priori based on clinical and oncological relevance rather than by stepwise statistical selection, and with consideration of events-per-variable constraints to avoid model overfitting. Survival was presented as hazard ratio (HR) with corresponding 95% confidence interval (CI). The proportional hazards assumption was formally assessed for all variables included in the Cox models using Schoenfeld residuals, and no relevant violations were observed. Pairwise Cox proportional hazards regression analyses were conducted to evaluate the impact of postoperative complications and ICU readmission on overall survival. All statistical tests were two sided, with a threshold of significance set at a P value of less than 0.05.

## Results

### Overall study population

A total of 733 patients underwent IL-OE with curative intent for invasive adenocarcinoma (79.8%, *n* = 585) or squamous cell carcinoma (20.1%, *n* = 147) during the examined study period. The mean post-surgical follow up was 27 months. Clinicopathological parameters of the overall cohort are presented in Table 1. The median age was 62 years. 82% (*n* = 601) were male and 18% (*n* = 132) female. The most prevalent comorbidities included hypertension (54.3%, *n* = 398), and diabetes mellitus type 2 (13.6%, *n* = 100) (Table 1). The most often performed surgical procedure was a hybrid approach combining abdominal laparoscopy and right antero-lateral thoracotomy (69.2%, *n* = 507), followed by total minimally invasive surgery (21.3%, *n* = 156). Additionally, 9.5% (*n* = 70) of patients underwent an open operation. The majority received a neoadjuvant treatment (91.7%, *n* = 672) with 67.6% (*n* = 454) undergoing a neoadjuvant radiochemotherapy and 30.1% (*n* = 202) receiving perioperative chemotherapy. The most common pathological T stage was pT3 (46.1%, *n* = 338), followed by pT1 (18.4%, *n* = 135), pT0 (17.6%, *n* = 129), pT2 (15.4%, *n* = 113) and pT4 (2.5%, *n* = 18).

**Table 1:** Comparison of clinicopathological parameters by ICU readmission

Characteristics	Overall *n* = 733 (%)	no reaICU *n* = 636 (%)	reaICU *n* = 95 (%)	*p* value
Age at presentation (years, mean)	62	62	62	NA
Sex				
Male	601 (82)	520 (81.8)	79 (83.2)	0.8524
Female	132 (18)	116 (18.2)	16 (16.8)	
BMI (kg/m^2^, mean)				
< 30	561 (76.5)	488 (76.7)	72 (75.8)	0.8676
≥ 30	171 (23.2)	148 (23.3)	23 (24.2)	
ASA				
I	88 (12)	76 (11.9)	11 (11.6)	
II	399 (54.4)	348 (54.7)	50 (52.6)	
III	243 (33.2)	219 (34.4)	34 (35.8)	0.9540
IV	2 (0.3)	2 (0.3)	0 (0)	
Comorbidities				
Hypertension	398 (54.3)	341 (53.6)	56 (58.9)	
COPD	65 (8.9)	56 (8.8)	9 (9.5)	0.406
Diabetes	100 (13.6)	90 (14.1)	11 (11.6)	
Renal disease	36 (4.9)	29 (4.6)	7 (7.4)	
Histology				
Adenocarcinoma	585 (79.8)	515 (81)	68 (71.6)	0.1334
Squamous cell carcinoma	147 (20.1)	120 (18.9)	27 (28.4)	
Neoadjuvant therapy regimen				
None	61 (8.3)	50 (7.9)	11 (11.6)	
RCT (CROSS)	454 (67.6)	399 (62.7)	54 (56.8)	0.8751
CT (FLOT)	202 (30.1)	173 (27.2)	28 (29.5)	
others	13 (1.9)	11 (1.7)	2 (2.1)	
Pathological T stage				
pT0	129 (17.6)	111 (17.5)	18 (18.9)	
pT1	129 (17.6)	120 (18.9)	15 (15.7)	0.8463
pT2	113 (15.4)	101 (15.9)	12 (12.6)	
pT3	338 (46.1)	290 (45.6)	46 (48.4)	
pT4	18 (2.5)	14 (2.2)	4 (4.2)	
Pathological N stage				
pN0	378 (51.6)	335 (52.7)	43 (45.3)	0.158
pN+	355 (48.4)	301 (47.3)	52 (54.7)	
Surgical procedure				
Hybrid	507 (69.2)	440 (69.1)	65 (68.4)	
Total MIC	156 (21.3)	137 (21.5)	19 (20)	0.9619
Open	70 (9.5)	59 (9.3)	11 (11.6)	
ICU length of stay (days)				
1–2	400 (54.6)	394 (61.9)	5 (5.3)	**< 0.0001**
> 2	330 (45)	242 (38.1)	90 (94.7)	

Overall patient cohort was divided into two groups based on readmission to ICU. Values represent patient numbers and percentages unlike indicated otherwise. Bold indicates significant findings

*No reaICU* no readmission to ICU, *reaICU* readmission to ICU, *BMI* Body mass index, *RCT* Radiochemotherapy, *CT* Chemotherapy, *MIC* Minimal invasive surgery

### ICU readmission and clinicopathological characteristics

For a more in-depth analysis of the impact of ICU readmission during the postoperative period, patients were divided into two groups: those without ICU readmission (no reaICU) and those with ICU readmission (reaICU) (Table 1). Comparing the comorbidity profiles between the reaICU and no-reaICU cohorts revealed no significant differences. Similarly, the ASA values were equivalent. The histological subtype had no impact on the postoperative course, irrespective of ICU readmission (Table S1). Furthermore, it showed no effect on overall survival (*p* = 0.98 h 1, 95% CI 0.72–1.4) (Fig. S2). In the reaICU cohort, 94.7% (*n* = 90) required an ICU stay exceeding 2 days, while in the no-reaICU cohort, over half of the patients (54.6%, *n* = 400) had an ICU stay limited to 1–2 days (Table 1).

### Complications and overall survival

**Fig. 1:**
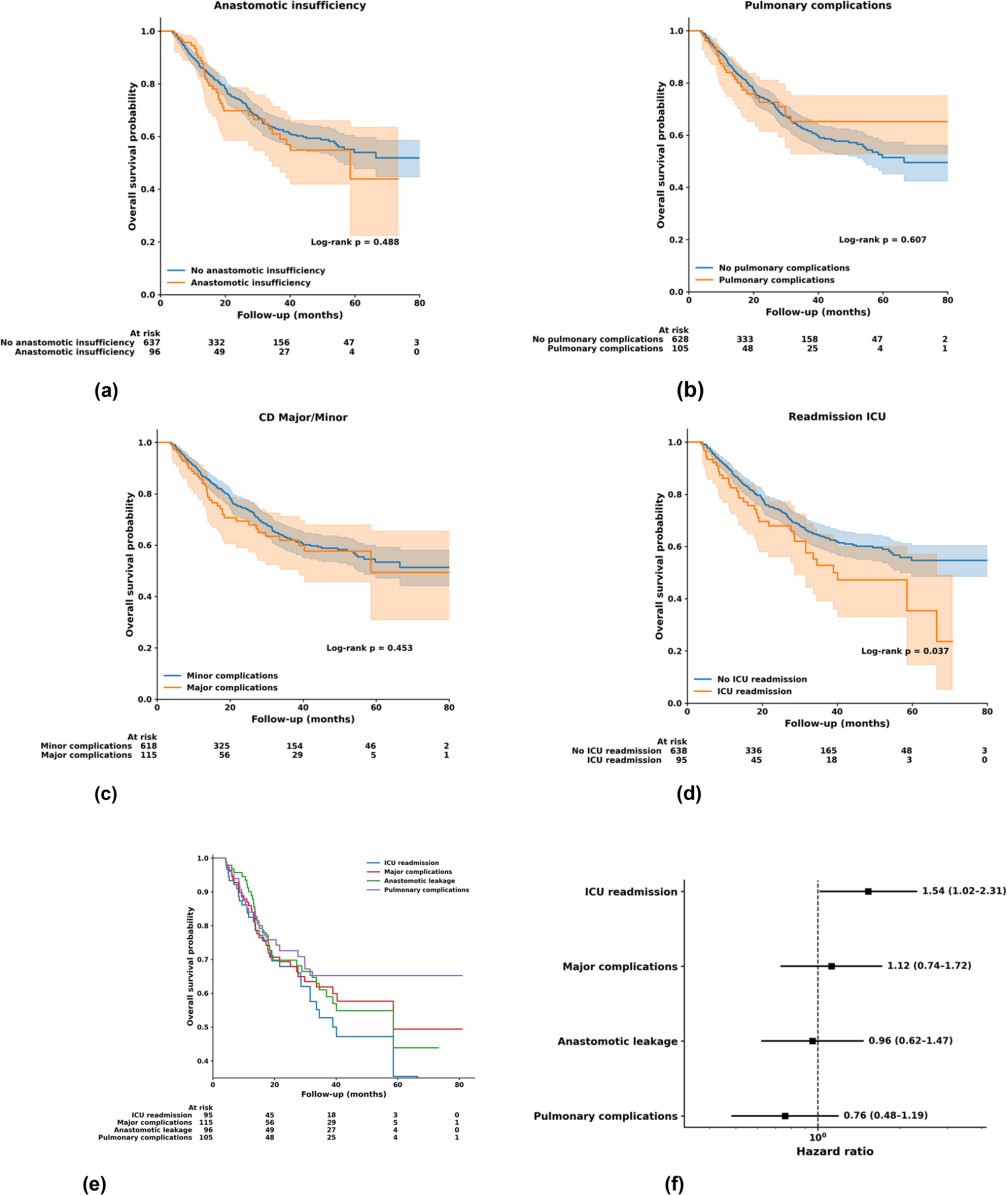
Univariate analysis of the impact of postoperative complications and ICU readmission on overall survival after Ivor Lewis esophagectomy. Kaplan–Meier survival curves illustrating overall survival stratified by (**a**) anastomotic insufficiency, **b** pulmonary complications, **c** complication severity according to Clavien–Dindo classification (minor vs. major), and **d** postoperative ICU readmission. Shaded areas represent 95% confidence intervals. Survival differences between groups were assessed using the log-rank test, with corresponding p-values indicated in each panel. **e** Combined Kaplan–Meier analysis comparing overall survival across patients with ICU readmission, major complications, anastomotic leakage, and pulmonary complications. **f** Forest plot derived from univariate Cox proportional hazards regression analysis showing hazard ratios (HRs) with 95% confidence intervals for the association of each variable with overall survival. Only ICU readmission was significantly associated with reduced overall survival (HR 1.54, 95% CI 1.02–2.31)

**Fig. 2:**
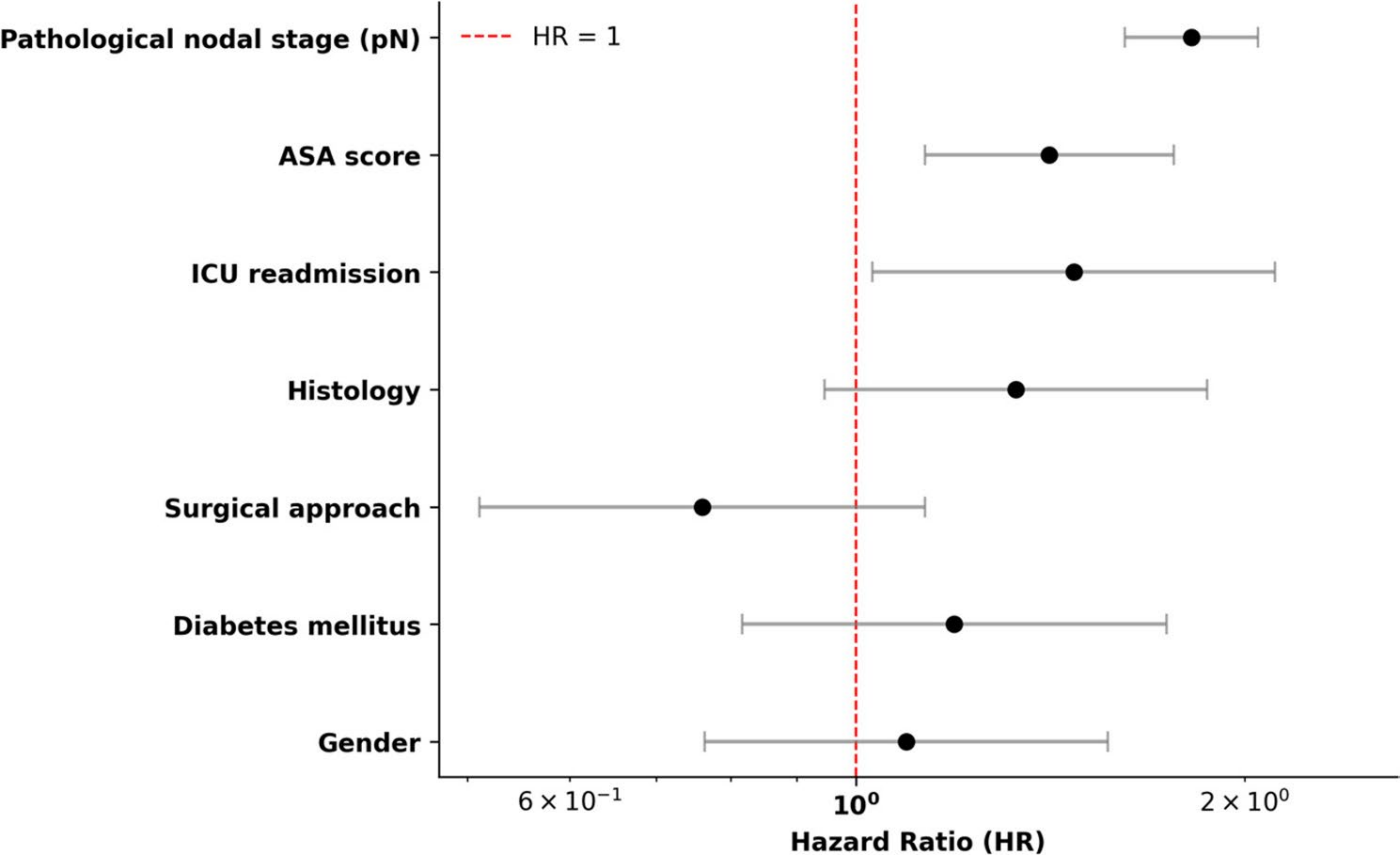
Adjusted effect of ICU readmission on overall survival. Forest plot illustrating hazard ratios (HRs) with 95% confidence intervals (CIs) from a multivariable Cox proportional hazards model assessing the association between ICU readmission and overall survival. The model was adjusted for gender, pathological nodal stage (pN), ASA score, histology, surgical approach, and diabetes mellitus. The dashed vertical line indicates the line of no effect (HR = 1). ICU readmission remained independently associated with reduced overall survival after adjustment for relevant clinicopathological covariates

Postoperative complications were reported in 63.1% (*n* = 463) of patients in the overall cohort (Table 2). 84.3% (*n* = 618) experienced only minor complications (Clavien-Dindo < IIIb), and 36.7% of these (*n* = 269) had an entirely unremarkable postoperative course (CD 0). AL was diagnosed in 13.1% (*n* = 96) of patients, with the majority (61.4%, *n* = 59) managed primarily through interventional treatments such as endoVAC and/or stent therapy. 14.3% (*n* = 105) of patients underwent pulmonary complications, predominantly pneumonia and insertion of a pleural effusion drainage. Neither major complications (Clavien–Dindo ≥ IIIb; *p* = 0.453, HR 1.12, 95% CI 0.74–1.72), anastomotic leakage (*p* = 0.488, HR 0.96, 95% CI 0.62–1.47), nor pulmonary complications (*p* = 0.607, HR 0.76, 95% CI 0.48–1.19) were associated with impaired overall survival in univariate analysis (Fig. 1). Solely the ICU readmission rate (13%, *n* = 95) had a significant impact on survival probability (*p* = 0.037, HR 1.54, 95% CI 1.02–2.31). Thus, to determine whether ICU readmission independently affects overall survival, a multivariable Cox proportional hazards regression model was performed adjusting for clinically and oncologically relevant covariates, including gender, pathological nodal stage (pN), ASA score, histological subtype, surgical approach, and diabetes mellitus (Fig. 2; Table 2). After adjustment, ICU readmission remained significantly associated with reduced overall survival (*p* = 0.034, HR 1.47, CI 95% 1.03–2.11), indicating that patients requiring postoperative ICU readmission had a substantially increased risk of death independent of tumor-related and patient-related factors. Among the covariates included in the model, N+ stage was strongly associated with worse survival (p = < 0.001, HR 1.81, CI 95% 1.61–2.04), confirming its role as a key oncological prognostic factor. Higher ASA scores were likewise associated with impaired survival (*p* = 0.002, HR 1.41, 95% 1.13–1.76), reflecting the impact of baseline patient condition. In contrast, histological subtype, surgical approach, diabetes mellitus, and gender did not show a significant independent association with overall survival. These findings demonstrate that ICU readmission represents an independent prognostic factor beyond established tumor and patient characteristics, suggesting that postoperative clinical deterioration severe enough to require ICU readmission has a lasting impact on long-term outcomes.

**Table 2:** Multivariable Cox regression analysis evaluating the impact of ICU readmission on overall survival

	HR	Lower confidence limit	Upper confidence limit	*p* value
reaICU vs. no reaICU	1.47	1.03	2.11	0.034
Male vs. Female	1.09	0.76	1.56	0.624
N0 vs. N+	1.81	1.61	2.04	**< 0.001**
Diabetes mellitus Yes vs. No	1.19	0.81	1.73	0.364
Histology SCC vs. AC	1.32	0.94	1.87	0.102
Surgical approach MIE vs. OE	0.76	0.51	1.13	0.176
ASA score	1.41	1.13	1.76	**0.002**

Results of a multivariable Cox proportional hazards model assessing the independent effect of ICU readmission on overall survival, adjusted for gender, pathological nodal stage (pN), ASA score, histology, surgical approach, and diabetes mellitus. Data are presented as hazard ratios (HRs) with 95% confidence intervals (Cis) and corresponding *p*-values. reaICU readmission to ICU, N+ defined as N1, N2 and N3, SCC squamous cell carcinoma, AC adenocarcinoma, OE open esophagectomy, MIE minimal invasive esophagectomy

### Association of ICU readmission, complications and survival

Major complications occurred in 54.7% of patients (*n* = 52) within the reaICU cohort (Table 3). Among these, 42.1% (*n* = 40) had pulmonary complications, 43.2% (*n* = 41) had an anastomotic leak – most of which were treated with endoscopic vacuum therapy – and 36.8% (*n* = 35) experienced atrial fibrillation. In contrast, 9.9% (*n* = 63) of patients being not readmitted to ICU experienced major complications. Among the no reaICU cohort, 8.6% (*n* = 55) underwent an anastomotic leakage, 7.1% (*n* = 45) developed an atrial fibrillation and 10.1% (*n* = 64) had pulmonary complications.

**Table 3:** Postoperative complications based on ICU readmission.

Complications	Overall *n* = 733 (%)	No reaICU *n* = 636 (%)	reaICU *n* = 95 (%)	*p* value
Clavien-Dindo				
I	269 (36.7)	266 (41.8)	2 (2.1)	
II	28 (3.8)	26 (4.1)	2 (2.1)	
IIIIIa	67 (9.1)	58 (9.1)	9 (9.5)	
IIIb	253 (34.5)	223 (35.1)	29 (30.5)	**< 0.0001**
IVa	53 (7.2)41 (5.6)	35 (5.5) 19 (3)	18 (18.9) 22 (23.2)	
IVb	19 (2.6)	9 (1.4)	10 (10.5)	
V	2 (0.3)	0 (0)	2 (2.1)	
Clavien-Dindo minor/major				
CD < IIIb	618 (84.3)	573 (90.1)	43 (45.3)	**< 0.0001**
CD ≥ IIIb	115 (15.7)	63 (9.9)	52 (54.7)	
Anastomotic leakage	96 (13.1)	55 (8.6)	41 (43.2)	
ECCG Type I	2 (2.1)	1 (1.8)	1 (2.5)	
ECCG Type II	62 (64.6)	37 (67.3)	25 (62.5)	
ECCG Type III	32 (33.3)	17 (30.9)	14 (35)	**< 0.0001**
Anastomotic leakage management				
Conservative	2 (2.1)	1 (1.8)	1 (2.4)	
EndoVAC	39 (40.6)	24 (43.6)	16 (39)	
Stent	3 (3.1)	2 (3.6)	1 (2.4)	
EndoVAC + Stent	20 (20.8)	13 (23.6)	7 (17.1)	0.8429
Reoperation	8 (8.3)	4 (7.3)	3 (7.3)	
endoVAC/Stent + Reoperation	24 (25)	11 (20)	13 (31.7)	
Pulmonary complications	105 (14.3)	64 (10.1)	40 (42.1)	
Pleural effusion drainage	91 (12.4)	60 (9.4)	30 (31.6)	
Pneumonia	77 (10.5)	46 (7.2)	30 (31.6)	
Resp. insufficiency with ventilation > 72 h	43 (5.9)	26 (4.1)	17 (17.9)	**< 0.0001**
Tracheostomy	22 (3)	10 (1.6)	12 (12.6)	
Atelectasis with bronchoscopy	13 (1.8)	10 (1.6)	3 (3.2)	
Atrial fibrillation	81 (11.1)	45 (7.1)	35 (36.8)	**< 0.0001**
DGCE	191 (26.1)	167 (26.3)	24 (25.3)	0.8369
Recurrent nerve injury	11 (11.5)	11 (1.7)	0 (0)	NA
Chylothorax	11 (1.5)	6 (0.9)	5 (5.3)	**0.0013**

Overall patient cohort was divided into two groups based on readmission to ICU. Values represent patient numbers and percentages unlike indicated otherwise. Bold indicates significant findings

*No reaICU* no readmission to ICU, *reaICU* readmission to ICU, *CD* Clavien-Dindo, *ECCG* Esophagectomy Complications Consensus Group, *DGCE* Delayed gastric conduit emptying

**Fig. 3:**
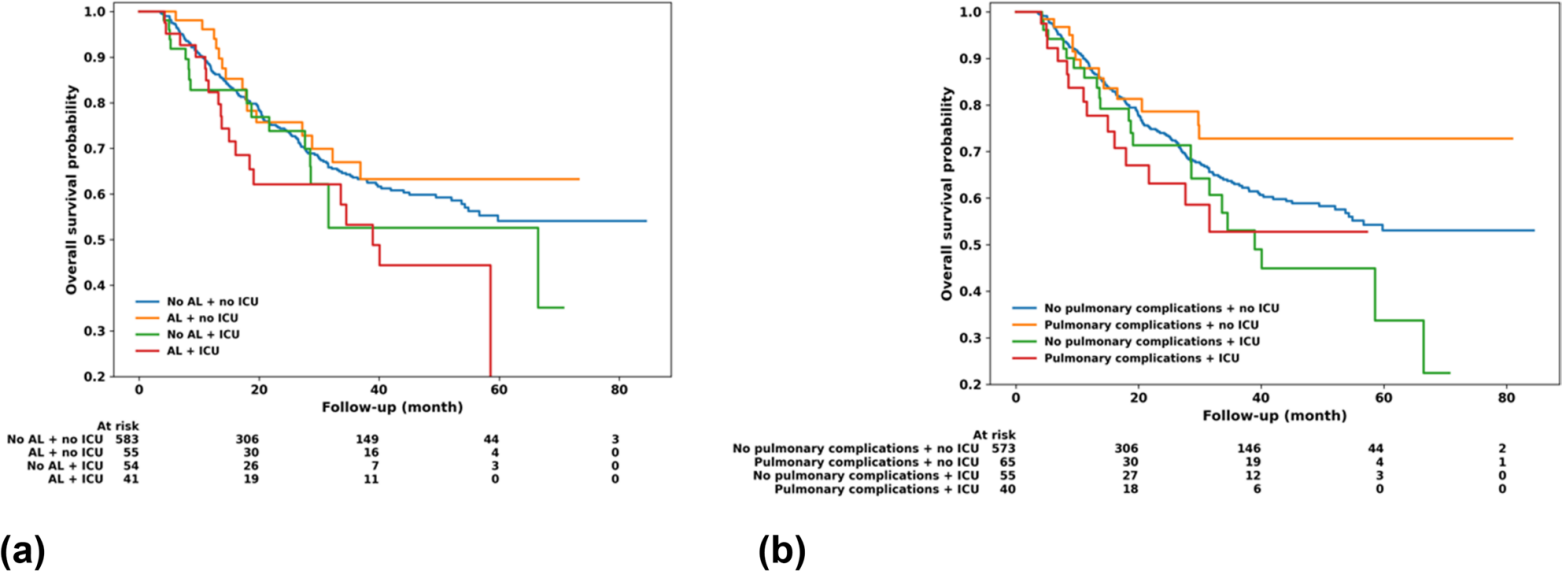
Combined impact of anastomotic leakage or pulmonary complications and ICU readmission on overall survival. **a** Kaplan–Meier curves depicting overall survival stratified by the presence of anastomotic leakage (AL) and postoperative ICU readmission. Patients were categorized into four groups: no AL without ICU readmission (No AL + no ICU), AL without ICU readmission (AL + no ICU), no AL with ICU readmission (No AL + ICU), and AL with ICU readmission (AL + ICU). **b** Kaplan–Meier curves illustrating overall survival stratified by the presence of pulmonary complications and postoperative ICU readmission. Patients were categorized into four groups: no pulmonary complications without ICU readmission, pulmonary complications without ICU readmission, no pulmonary complications with ICU readmission, and pulmonary complications with ICU readmission. Numbers at risk are displayed below the x-axis. Overall survival was defined from 90 days after surgery

To further investigate the combined impact of anastomotic leakage and ICU readmission on overall survival, patients were stratified into four groups based on the presence of AL and ICU readmission (Fig. 3a). Most patients (*n* = 583) had neither AL nor ICU readmission, while 55 patients experienced AL without ICU readmission, 54 patients required ICU readmission without AL, and 41 patients had both AL and ICU readmission.

Kaplan–Meier analysis demonstrated distinct survival patterns across the four groups. Patients without ICU readmission, regardless of the presence of AL, showed the most favorable survival outcomes. In contrast, patients requiring ICU readmission exhibited impaired overall survival, particularly when ICU readmission occurred in combination with anastomotic leakage.

These findings suggest that while anastomotic leakage alone does not substantially affect long-term survival, its combination with ICU readmission is borderline significant for impaired overall survival (*p* = 0.064, HR 1.9, 95% CI 0.96–3.75) (Table S3a). Same pattern applies for the combination of pulmonary complications and readmission to ICU (Fig. 3b). Pulmonary complications alone did not appear to substantially impair overall survival (Table S3b) (*p* = 0.183, HR 0.68, 95% CI 0.39–1.12). However, pulmonary complications in combination with readmission to ICU were borderline significant for worse overall survival (*p* = 0.085, HR 1.94, 95% CI 0.9–4.12). These findings suggest that the need for ICU readmission, rather than the occurrence of pulmonary complications or AL per se, is the key determinant of impaired long-term survival. This supports the hypothesis that not the complication itself, but rather the severity of the clinical course—reflected by the need for ICU readmission—plays a decisive role in long-term prognosis.

## Discussion

A decisive question in esophageal cancer management is whether mitigating short-term postoperative complications can improve long-term survival rates. While the existing data on this subject remains inconclusive, numerous studies have postulated a potential link between postoperative complications and reduced long-term survival [4, 21, 22]. However, our analysis of a large, highly standardized cohort in a very high-volume center over a six-year period challenges this hypothesis. Specifically, we found no significant impact of anastomotic leakage (AL) or pulmonary complications (PC) on the long-term survival of esophageal cancer patients (Fig. 1a, b). The results of the current study are in accord with previously published data showing no correlation between perioperative morbidity and long-term survival following esophagectomy [6, 7, 23, 24]. Notably, in our cohort, most patients underwent hybrid esophagectomy (69.2%), which differs fro studies focusing on fully open or totally minimally invasive approaches. Given that hybrid procedures are associated with lower pulmonary complication rates while maintaining comparable oncologic outcomes, this may have influenced both complication patterns and the missing impact on overall survival and should be considered when comparing our results with previous studies.

Patients readmitted to the ICU during their postoperative stay had a significantly lower survival probability in both univariate and multivariable analysis (*p* = 0.037, HR 1.54, 95% CI 1.02–2.31 (Fig. 1d, f); *p* = 0.034, HR 1.47, 95% CI 1.03–2.11) (Table 3)). Furthermore, while anastomotic leakage and pulmonary complications generally had no significant effect on long-term survival, readmission to the ICU while experiencing an anastomotic leak or pulmonary complication had more likely a worse outcome (Fig. 3). After two years, 47.2% of non-readmitted patients were alive, compared to an overall survival rate of 42.1% in the ICU readmission cohort. While baseline characteristics such as ASA score and comorbidity profiles appeared comparable between groups in univariate analyses, the multivariable Cox regression showed that a higher ASA score was independently associated with worse overall survival (*p* = 0.002, HR 1.41, 95% CI 1.13–1.76) indicating that preexisting comorbidity burden does contribute to long-term outcomes. However, ICU readmission remained associated with impaired survival after adjustment (Fig. 2), suggesting that its effect cannot be fully explained by baseline patient characteristics alone. These results indicate that both preoperative patient condition and postoperative complications requiring ICU readmission independently impact long-term survival. Thus, the reduced survival observed in the ICU readmission cohort is likely driven by a combination of baseline vulnerability (ASA score) and severity of postoperative clinical deterioration, rather than comorbidities alone.

One possible explanation is that ICU readmission leads to a significantly prolonged hospital stay, delaying or precluding adjuvant cancer treatment and potentially increasing recurrence rates. After IL-OE, patients typically experience a significant but self-limiting systemic inflammatory response if their postoperative course is uncomplicated. However, those readmitted to the ICU often meet the criteria for sepsis due to complications such as AL or pneumonia. This creates an additional burden, first from the surgical procedure itself and secondary from the ensuing complications [25]. This results in an extensive release of pro-inflammatory cytokines and interleukins leading to immunosuppression and a pro-inflammatory microenvironment promoting local cancer recurrence and exacerbating the metastatic process [6, 26, 27]. Therefore, a higher recurrence rate provoked by complications may be a contributing factor to poorer long-term survival. However, ICU readmission could also be interpreted as an indicator of a complicated postoperative trajectory rather than a direct causal determinant of impaired survival. Furthermore, ICU readmission may also be influenced by institutional practices.

Surprisingly, major complications did not substantially affect long term survival (Fig. 1c), despite the reaICU cohort presents with a major complication rate of 54.7% (*n* = 52) compared to just 9.9% (*n* = 63) in the no reaICU cohort (Table 2). 96 patients from the overall cohort of 733 developed an anastomotic leak that needed either interventional or operative treatment. However, 57.3% (*n* = 55) of patients treated for AL did not require ICU readmission. One possible explanation for the missing effect of postoperative AL on the long-term survival might be due to its highly sufficient and timely management in a high-volume center that progressively avoids requirement of intensive care treatment.

There is conflicting evidence on the impact of anastomotic leakage on long-term survival following esophagectomy for esophageal cancer. A crucial factor is the volume effect, accentuating the influence of surgical center experience on outcomes. This is highlighted by a meta-analysis by Gujjuri et al. showing that AL had a significant impact on survival in low-volume centers but not high-volume centers [13]. This may be due to limited resources, such as endoscopic or radiologic capabilities and or experience in low-volume centers, hindering the effective treatment of AL. Consistent with this, Ghaferi et al. showed a higher failure-to-rescue rate in low volume centers with 30.3% copared to 13.1% inhigh-volume centers when it comes to management of postoperative complications [28]. Additionally, a meta-analysis including 13 studies demonstrated a significant survival benefit in favor of high-volume centers compared to their low-volume counterparts [29]. Consistent with this, Finlayson et al. reported an 8.5% higher diffeence in mortality rate, particularly among older patients, in low-volume centers compared to high-volume centers [30]. Whether and how optimized perioperative management in high-volume centres directly or indirectly impact on long-term survival has to be further elucidated.

Previous studies have identified PC as a primary contributor to postoperative morbidity in patients undergoing esophagectomy, with reported incidences of up to 31%. In contrast, our analysis did not demonstrate a statistically significant association between PC and long-term overall survival (*p* = 0.607). This finding may in part reflect limited statistical power, as PC occurred in only 14.3% (*n* = 105) of the overall cohort, potentially reducing the ability to detect small to moderate effects, particularly in subgroup analyses. However, a borderline significance was observed (*p* = 0.08) when readmitted to ICU suggesting that the impact of PC on survival is not uniform but depends on their severity and the overall postoperative course. The majority of PC in our cohort consisted of pneumonia and pleural effusions requiring drainage, whereas more severe conditions such as prolonged respiratory insufficiency or the need for tracheostomy were relatively infrequent. This distribution may attenuate the overall effect of PC on long-term survival.

Similarly, two studies examining patients who underwent either open transthoracic or hybrid esophagectomy reported no significant impact of PC on overall survival [31, 32]. Minimally invasive surgery was associated with a 50% lower risk of major pulmonary complications compared to open surgery [31]. Notably, in our analysed cohort, the majority of patients underwent at least hybrid minimally invasive surgery, with only 9.5% (*n* = 70) having an open IL-OE (Table 1), thereby reducing the overall risk of PC. This might explain in part the absence of a detrimental effect of PC on long-term survival. These findings align with the results of the TIME trial, which demonstrated that total minimally invasive esophagectomy was linked to a 70% lower incidence of pneumonia at two weeks compared to open esophagectomy [33]. The hypothesis that reducing PC and other complications by minimally invasive techniques results in longer oncologic survival has to be tested in prospective trials.

The heterogeneous data on postoperative complications and long-term outcomes might be a result of differences in institutional volume, heterogenous patient selection, varying follow-up periods, and lack of predefined definitions for complications [34]. To accurately assess the impact of various postoperative complications on long-term survival, a multicenter study involving high-volume centers with substantial expertise in esophagectomy and perioperative complication management is essential.

### Limitations

The present study has several limitations. First, its retrospective and monocentric design may limit the generalizability of the findings. Second, the relatively short follow-up period, with a mean duration of 27 months, falls short of the commonly accepted 5-year benchmark for assessing long-term oncologic outcomes after esophagectomy. Consequently, the present findings should primarily be interpreted as reflecting mid-term overall survival. It cannot be excluded that potential differences in late cancer-related mortality may not yet be fully captured within this timeframe. Therefore, extended follow-up of this cohort will be necessary to determine whether survival differences emerge over time. Additionally, although a 90-day landmark analysis was performed to reduce bias from early postoperative mortality, complications occurring after early discharge may still be underreported due to the retrospective study design.

## Conclusion

IL-OE remains one of the most complex surgical procedures. While in-hospital mortality rates declined during the last decades, postoperative complications remain a significant concern. However, the analysis of this highly standardized patient cohort regarding the most prevalent complications such as anastomotic leakage and pulmonary complications, did not reveal an impact on survival. Instead, ICU readmission during the postoperative course significantly impacted on long-term oncologic outcome. Efforts should be prioritized towards early detection and treatment of postoperative complications to reduce the risk of severe secondary complications requiring ICU readmission. The underlying cause or mechanism how ICU readmission after esophagectomy effects survival must be further elucidated in clinical trials and translational studies.

## Supplementary Information

Supplementary Material 1.

## Data Availability

The authors confirm that the data supporting this study’s findings are available within the article and/or its supplementary materials. The datasets used and/or analysed during the current study are available from the corresponding author on reasonable request.
