# Supplementary material for: Complications and survival after Ivor Lewis esophagectomy
Source: BMC Cancer. 2026 Jul 1;26:802. doi: 10.1186/s12885-026-16447-8 (PMC13326540; doi:10.1186/s12885-026-16447-8)
Supplement: Supplementary file 1 — Supplementary Material 1. [file 12885_2026_16447_MOESM1_ESM.docx]

**Complications and survival after Ivor-Lewis esophagectomy**

Abu Hejleh A.^1^, Lemties J.^1^, Kreutzer I.^1^, Wirsik N.M.^1^, Krauss D.T.^1^, Torabi S.^2^, Jung J-O.^1^, Schröder W.^1,3^, Schlößer H. A.^1^, Schmidt T.^1^, Fuchs H.F.^1^, Bruns C.J.^1^, Schiffmann L.M.^1^

^1^ Department of General, Visceral, Thoracic and Transplantation Surgery, Faculty of Medicine and University Hospital Cologne, University of Cologne, Cologne, Germany.

^2^ Department of Anesthesiology and Intensive Care Medicine, Medical Faculty of Cologne University, University Hospital of Cologne, Cologne, Germany.

^3^ Department of General, Visceral and Oncological Surgery, University Hospital Wuppertal, University of Witten-Herdecke, Wuppertal, Germany.

**Corresponding author: Schiffmann L.M.**

**Supplementary Materials - Index**

| **Supplementary Results** |  |
| --- | --- |
| Table S1 | *pag.5* |
| Figure S2 | *pag. 5* |
| Table S3 | *pag. 11* |
| **Supplementary Figures and Tables** |  |
| Table S1 | *pag. 21* |
| Figure S2 | *pag. 22* |
| Table S3 | *pag. 22/23* |

**Supplementary Figures and Tables**

| **Complications** | **AEG n (%)** | **PEC n (%)** | ***p* value** |
| --- | --- | --- | --- |
| **Total** | 585 | 147 |  |
| **Clavien-Dindo**  0  I  II  IIIa  IIIb Iva  Ivb  V | 222 (37.9)  22 (3.8)  47 (8)  209 (35.7)  40 (6.8)  32 (5.5)  11 (1.9)  1 (0.2) | 47 (32)  6 (4.1)  20 (13.6)  44 (29.9)  12 (8.2)  9 (6.1)  8 (5.4)  1 (0.7) | 0.1791  0.8561  **0.0363**  0.1866  0.5759  0.7585  0.0152  0.2902 |
| **Clavien-Dindo minor/major**  CD < IIIb  CD > IIIb | 501 (85.6)  84 (14.4) | 117 (79.6)  30 (20.4) | 0.6831 |
| **Anastomotic leakage**  ECCG Type I  ECCG Type II  ECCG Type III  **Anastomotic leakage management**  Conservative  EndoVAC  Stent  EndoVAC + Stent  Reoperation  endoVAC/Stent + Reoperation | 71 (12.1)  1 (1.4)  46 (64.8)  24 (33.8)  1 (1.4)  30 (42.3)  2 (2.8)  15 (21.1)  5 (7)  18 (25.4) | 25 (17)  1 (4)  16 (64)  8 (32)  1 (4)  10 (40)  1 (4)  5 (20)  2 (8)  6 (24) | 0.1179  0.4353  0.9435  0.8694  0.4353  0.8442  0.77  0.905  0.8741  0.8932 |
| **Pulmonary complications**  Pleural effusion drainage  Pneumonia  Resp. insufficiency with ventilation >72h  Tracheostomy  Atelectasis with bronchoscopy | 81 (13.8)  74 (12.6)  58 (9.9)  33 (5.6)  16 (2.7)  8 (1.4) | 23 (15.6)  17 (11.6)  18 (12.2)  10 (6.8)  6 (4.1)  4 (2.7) | 0.5763  0.7215  0.4076  0.5923  0.3926  0.2479 |
| **Atrial fibrillation** | 60 (10.3) | 21 (14.3) | 0.1639 |
| **DGCE** | 156 (26.7) | 35 (23.8) | 0.4807 |
| **Recurrent nerve injury** | 5 (0.85) | 6 (4.1) | **0.004** |
| **Chylothorax** | 9 (1.5) | 2 (1.4) | 0.8741 |

**Table S1:** Overall patient cohort was divided into two groups based on histological type. Values represent patient numbers and percentages unlike indicated otherwise.

**
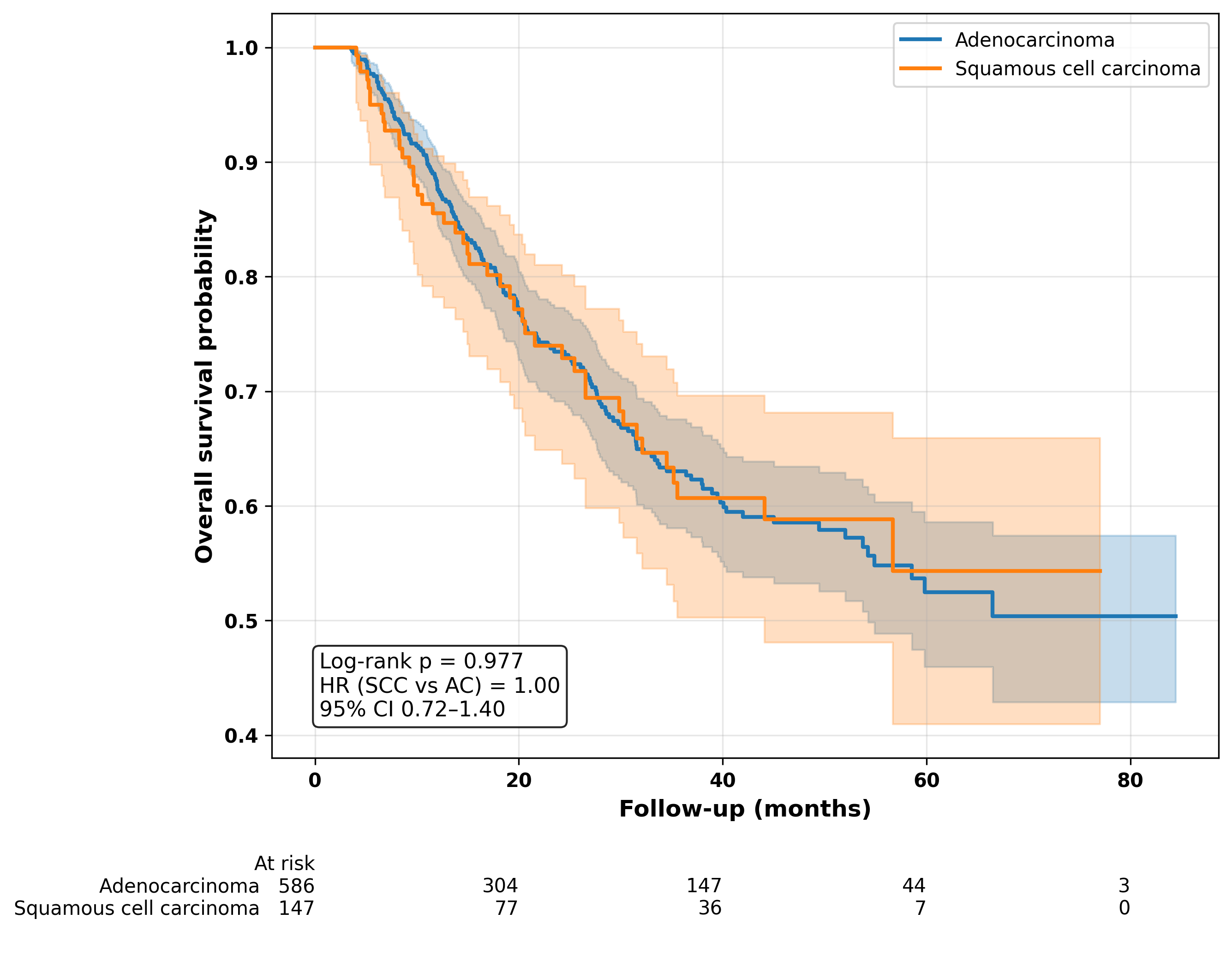
**

**Figure S2:** Kaplan-Meier survival curve illustrating overall survival of patients undergoing esophagectomy for esophageal cancer regarding histological type. AC adenocarcinoma, SCC squamous cell carcinoma.

| **Reference group** | **Comparison group** | **HR** | **CI_lower_95** | **CI_upper_95** | **p_value** |
| --- | --- | --- | --- | --- | --- |
| No AL + no reaICU | AL + no reaICU | 0.86 | 0.5 | 1.45 | 0.563 |
| No AL + no reaICU | No AL + reaICU | 1.27 | 0.77 | 2.1 | 0.343 |
| No AL + no reaICU | AL + reaICU | 1.62 | 1.01 | 2.61 | 0.046 |
| AL + no reaICU | No AL + reaICU | 1.52 | 0.76 | 3.06 | 0.236 |
| AL + no reaICU | AL + reaICU | 1.90 | 0.96 | 3.75 | 0.064 |
| No AL + reaICU | AL + reaICU | 1.30 | 0.67 | 2.54 | 0.441 |

**(a)**

| **Reference group** | **Comparison group** | **HR** | **CI_lower_95** | **CI_upper_95** | **p_value** |
| --- | --- | --- | --- | --- | --- |
| No pul comp + no reaICU | pul comp + no reaICU | 0.68 | 0.39 | 1.2 | 0.183 |
| No pul comp + no reaICU | No pul comp + reaICU | 1.42 | 0.91 | 2.21 | 0.123 |
| No pul comp + no reaICU | pul comp + reaICU | 1.40 | 0.81 | 2.42 | 0.225 |
| pul comp + no reaICU | pul comp + reaICU | 2.08 | 1.05 | 4.13 | 0.037 |
| pul comp + no reaICU | pul comp + reaICU | 1.94 | 0.91 | 4.14 | 0.085 |
| No pul comp + reaICU | pul comp + reaICU | 1.08 | 0.55 | 2.15 | 0.818 |

**(b)**

**Table S3: Pairwise Cox regression analyses of overall survival according to anastomotic leakage or pulmonary complications and ICU readmission**

(a) Anastomotic leakage (AL): Patients were stratified into four groups based on the presence or absence of anastomotic leakage and ICU readmission: no AL + no ICU readmission, AL + no ICU readmission, no AL + ICU readmission, and AL + ICU readmission. Pairwise comparisons between all groups are presented as hazard ratios (HRs) with 95% confidence intervals (CIs) and corresponding p-values. (b) Pulmonary complications (pul comp): Patients were categorized into no pul comp + no ICU readmission, pul comp + no ICU readmission, no pul comp + ICU readmission, and pul comp + ICU readmission. Pairwise Cox regression results are shown as HRs with 95% CIs and p-values for all group comparisons.
